# Prior Knowledge Facilitates Mutual Gaze Convergence and Head Nodding Synchrony in Face-to-face Communication

**DOI:** 10.1038/srep38261

**Published:** 2016-12-02

**Authors:** C. Thepsoonthorn, T. Yokozuka, S. Miura, K. Ogawa, Y. Miyake

**Affiliations:** 1 Department of Computational Intelligence and Systems Science, Tokyo Institute of Technology, Yokohama, Japan

## Abstract

As prior knowledge is claimed to be an essential key to achieve effective education, we are interested in exploring whether prior knowledge enhances communication effectiveness. To demonstrate the effects of prior knowledge, mutual gaze convergence and head nodding synchrony are observed as indicators of communication effectiveness. We conducted an experiment on lecture task between lecturer and student under 2 conditions: prior knowledge and non-prior knowledge. The students in prior knowledge condition were provided the basic information about the lecture content and were assessed their understanding by the experimenter before starting the lecture while the students in non-prior knowledge had none. The result shows that the interaction in prior knowledge condition establishes significantly higher mutual gaze convergence (t(15.03) = 6.72, p < 0.0001; α = 0.05, n = 20) and head nodding synchrony (t(16.67) = 1.83, p = 0.04; α = 0.05, n = 19) compared to non-prior knowledge condition. This study reveals that prior knowledge facilitates mutual gaze convergence and head nodding synchrony. Furthermore, the interaction with and without prior knowledge can be evaluated by measuring or observing mutual gaze convergence and head nodding synchrony.

Behavioral neuroscience studies have demonstrated evidences on the impact of prior knowledge in learning and education in terms of memory^1^,^2^,^3^, comprehension^4^, and congruency effect, which enhances the specific brain areas connectivity^5^,^6^. Its influence is further implied through numerous educational studies where prior knowledge is believed to be the essential key that influences and assists the listener to create the linkage between the existing knowledge and the new information in order to have an effective learning^7^,^8^,^9^,^10^,^11^. The influences of prior knowledge in education have been widely investigated and the relationship of prior knowledge and interest^12^,^13^, student’s learnability and attentiveness^14^ has been found. Here, we can define that one who has prior knowledge is at “Ready State”: the internal state where one has acquired the required knowledge to accumulate and comprehend new knowledge or more advanced topics to be able to create knowledge linkage with the existing one. The previous studies from both behavioral neuroscience and education research fields provided evidences and suggested that prior knowledge enhances and has impact on learning activities effectiveness and success.

In communication research viewpoint, learning activities, such as teaching and lecturing, are also considered as a part of face-to-face and human-human interaction^15^,^16^,^17^. Many researchers have been studied the interaction between teacher and student to improve effectiveness and success in communication^18^,^19^,^20^,^21^. The indicators or evaluations on the effectiveness and success of the interaction can be assessed by various means, for instance, questionnaires^8^, clickers^22^, and interactional behaviors or nonverbal behaviors^23^,^24^,^25^. Observing interactional behaviors or nonverbal behaviors is one of the effective means to indicate the effectiveness and success in interaction since nonverbal behaviors and interactional behaviors are expressed unconsciously^26^,^27^, particularly, via mutual gaze convergence and head nodding synchrony. The relationship of prior knowledge and nonverbal-interactional behaviors can be investigated as one approach for evaluating the effectiveness and success in communication. However, such investigations are still limited.

This study aims to investigate the relationship between prior knowledge and nonverbal-interactional behaviors in face-to-face lecture task by observing the interaction between lecturer and student with/without prior knowledge: prior knowledge condition and non-prior knowledge condition. We observed mutual gaze convergence and head nodding synchrony as the representations of nonverbal-interactional behaviors and as the indicators of communication effectiveness. In order to further affirm the influence of prior knowledge toward mutual gaze convergence and head nodding synchrony, we divided the lecture content, which is used in both conditions, into 2 parts. Part 1 is highly related to prior knowledge and corresponds to the first half of the lecture content. Part 2 is rarely related to prior knowledge, corresponds to the second half of the lecture content, and is considered as baseline content because the content in Part 2 contains the information that is unfamiliar to the participants in both conditions. In this study, we firstly compared the total occurrence percentage of mutual gaze convergence and head nodding synchrony during the lecture as a whole between prior knowledge condition and non-prior knowledge condition. We also did the same comparison for Part 1 and Part 2 between the 2 conditions in order to emphasize the influence of prior knowledge. Moreover, we compared the difference between Part 1 and Part 2 in each condition and calculated the ratio of change in mutual gaze convergence and head nodding synchrony from Part 1 to Part 2 in each condition as well to affirm the effect of prior knowledge and the significance of the results. We hypothesize that prior knowledge has influence and facilitates nonverbal-interactional behaviors, namely mutual gaze convergence and head nodding synchrony. The difference in prior knowledge will be expressed differently via mutual gaze convergence and head nodding synchrony. And we can evaluate the interaction with or without prior knowledge by using mutual gaze convergence and head nodding synchrony as indicators.

## Results

**Figure 1:**
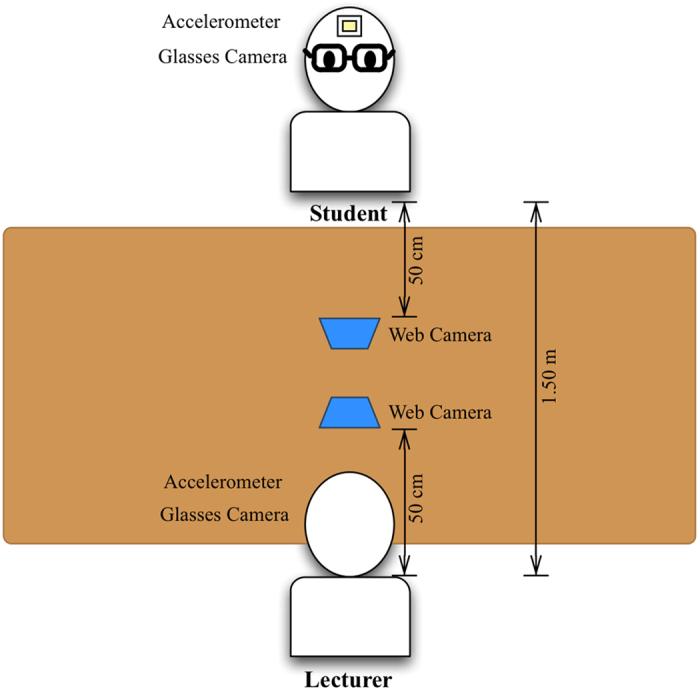
Experimental setup. The figure illustrates equipment setup and the position of the participants in each trial.

30 participants were grouped into 10 triplets and took the role of lecturer and student in one-to-one short lecture task. Each triplet consists of 2 pairs: a lecturer with a student with prior knowledge, and the same lecturer with a student with no prior knowledge (10 pairs in prior knowledge condition and 10 pairs in non-prior knowledge condition). In order to eliminate the order effect, we alternated trial order between prior knowledge condition and non-prior knowledge condition in each triplet. This experiment is also controlled to be within the attention span, which lasts for 10–20 minutes^28^. In each trial, the lecturer participant teaches the student participant using the same lecture content. Before the experiment, the students in prior knowledge condition were provided information related to the lecture content and assessed their understanding by the experimenter. This ensured that the students in prior knowledge condition were at Ready State before starting the lecture session. On the other hand, the students in non-prior knowledge condition had none. The lecture content is separated into 2 parts. Part 1 contains information that is highly related to the information provided to the students in prior knowledge condition in term of word choices and technical terms. Part 2 (baseline content) provides the information in the same main topic, however, rarely related to the information provided to the students in prior knowledge condition. During the experiment, the participants were seated facing directly to each other (Fig. 1). Each participant had to wear glasses camera and 2 web cameras were set in front of each participant to capture their gaze direction. In addition, 2 accelerometers were attached on each participant’s forehead for detecting their head nodding synchrony. The data from the first 30 seconds was deleted in order to exclude the initial attentiveness effect^29^.

### Mutual gaze convergence result

**Figure 2:**
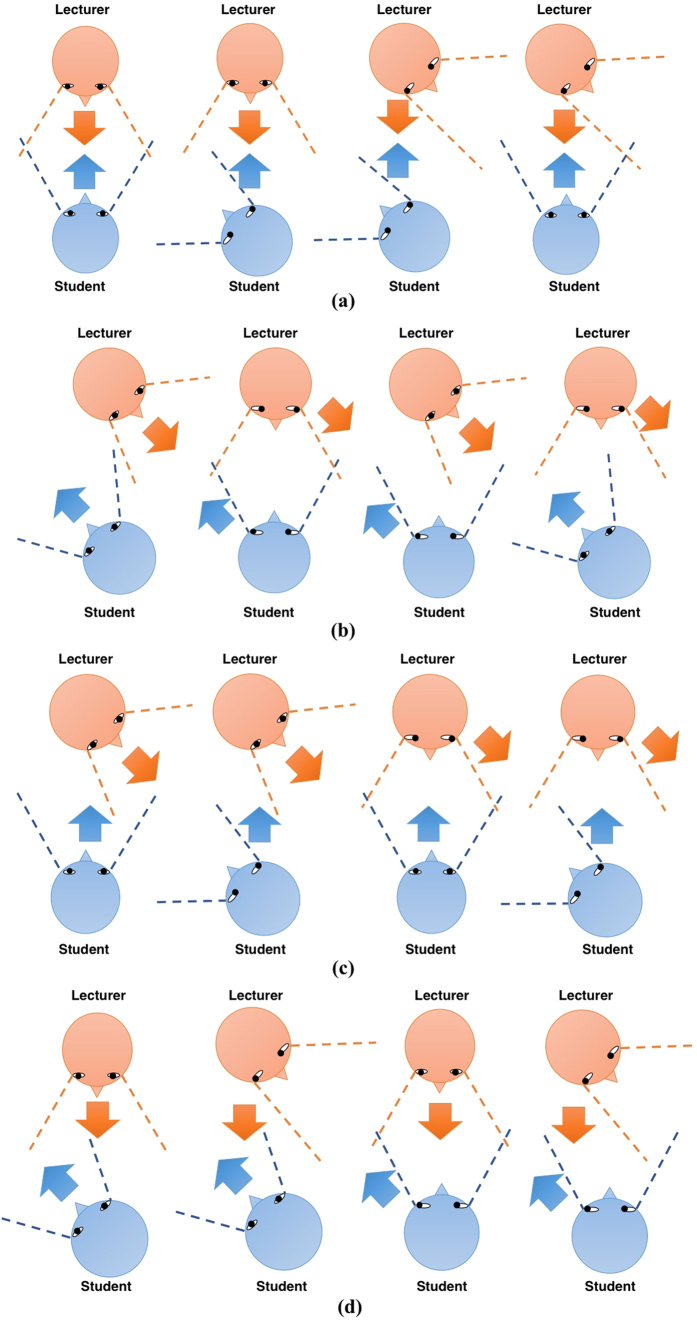
Types of gaze direction behavior. Dash lines represent head direction recorded via glasses cameras. Arrows represent gaze direction recorded via web cameras. **(a)** Possible scenarios of mutual gaze convergence. Both lecturer’s and student’s gaze directions are at each other. **(b)** Possible scenarios of non-mutual gaze convergence: 2-side averted. Both lecturer’s and student’s gaze directions are not at each other though there are some scenarios that their head directions are toward each other. **(c)** Possible scenarios of non-mutual gaze convergence: 1-side averted by lecturer. Lecturer’s gaze direction is not at the student while the student has straight gaze at the lecturer. **(d)** Possible scenarios of non-mutual gaze convergence: 1-side averted by student. Student’s gaze direction is not at the lecturer while the lecturer has straight gaze at the student.

In this study, we focus only on gaze direction instead of precise gaze position of the interactants. Mutual gaze convergence behavior is observed using 2 web cameras and 2 glasses cameras. The possible scenarios of mutual gaze convergence are shown in Fig. 2. We consider the interactants’ gaze behaviors as mutual gaze convergence when web cameras can capture looking straight gaze behavior to their partner from both individuals. Glasses cameras are used to confirm the individual’s head tilting direction (Fig. 2(a)). Other detected gaze behaviors, including both-side averted scenarios and one-side averted, are considered as non-mutual gaze convergence (Fig. 2(b–d)). We analyzed recorded data from 30 participants (10 pairs in prior knowledge condition and 10 pairs in non-prior knowledge condition, n = 20).

#### Total mutual gaze convergence (Total)

**Figure 3:**
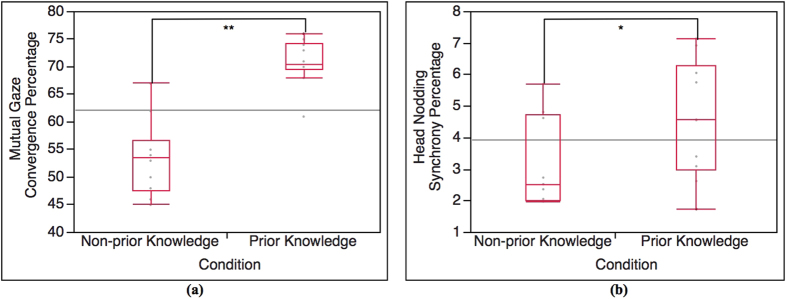
Analysis result of mutual gaze convergence and head nodding synchrony between prior knowledge condition and non-prior knowledge condition (Total). (**a**) T-test result of total mutual gaze convergence as a whole between prior knowledge condition and non-prior knowledge condition (n = 20, α = 0.05), t(15.03) = 6.72, p < 0.0001; highly significant. (**b**) T-test result of total head nodding synchrony as a whole between prior knowledge condition and non-prior knowledge condition (n = 19, α = 0.05), t(16.67) = 1.83, p = 0.04; significant).

We compared total mutual gaze convergence occurrence percentage during the interaction as a whole between prior knowledge condition and non-prior knowledge condition using T-test analysis (α = 0.05). The T-test result shows statistical significance in the occurrence percentage of total mutual gaze convergence between prior knowledge condition and non-prior knowledge condition as illustrated in Fig. 3(a), t(15.03) = 6.72, p < 0.0001. The result indicates that the interaction in prior knowledge condition establishes (highly) significantly higher mutual gaze convergence than the interaction in non-prior knowledge condition. The average percentages of total mutual gaze convergence of prior knowledge condition and non-prior knowledge condition are 70.80 and 53.50, respectively.

#### Mutual gaze convergence in Part 1 (Part 1)

**Figure 4:**
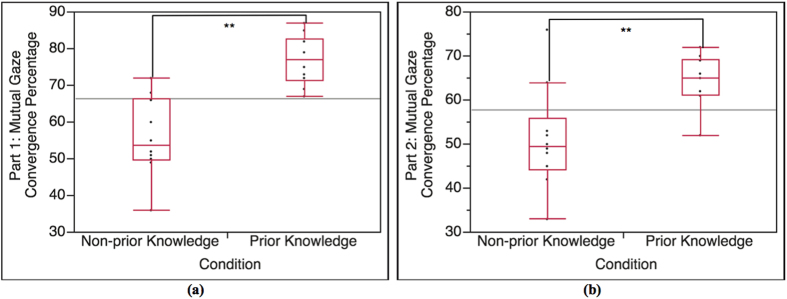
Analysis result of mutual gaze convergence for Part 1 and Part 2. **(a)** T-test result between Part 1 of prior knowledge condition and Part 1 of non-prior knowledge condition (n = 20, α = 0.05), t(15.08) = 5.21, p < 0.0001; highly significant. **(b)** T-test result between Part 2 of prior knowledge condition and Part 2 of non-prior knowledge condition (n = 20, α = 0.05), t(13.03) = 3.16, p = 0.003; highly significant.

The result data of each pair is divided into 2 parts according to the lecture content. We conducted cross-condition T-test analysis on the result in Part 1 (α = 0.05). The cross-condition T-test result of mutual gaze convergence percentage is shown in Fig. 4(a). According to the result, the occurrence percentage of mutual gaze convergence in Part 1 of prior knowledge condition is (highly) significantly higher than Part 1 of non-prior knowledge condition, t(15.08) = 5.21, p < 0.0001. The average percentages in Part 1 of prior knowledge condition and non-prior knowledge condition are 76.80 and 55.90, respectively.

#### Mutual gaze convergence in Part 2 (Part 2)

As Part 2 is considered as a baseline, we expected no statistical significance between the 2 conditions. However, the cross-condition T-test result shows that the mutual gaze convergence percentage in Part 2 of prior knowledge condition is (highly) significantly higher than Part 2 of non-prior knowledge condition, t(13.03) = 3.16, p = 0.003 (α = 0.05) (Fig. 4(b)). Though the statistical significance is observed, the result in Part 2 shows less statistical significance when compared to the result in Part 1. The average percentages in Part 2 of prior knowledge condition and non-prior knowledge condition are 64.30 and 51.20, respectively.

#### Mutual gaze convergence between Part 1 and Part 2 of each condition (Part 1-Part 2)

**Figure 5:**
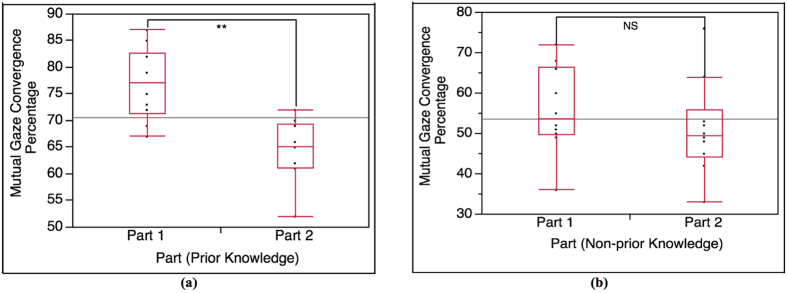
Analysis result of mutual gaze convergence occurrence percentage between Part 1 and Part 2 of each condition. **(a)** Paired T-test result of mutual gaze convergence occurrence percentage between Part 1 and Part 2 of prior knowledge condition (n = 20, α = 0.05), t(18) = 4.48, p < 0.0001; highly significant. **(b)** Paired T-test result of mutual gaze convergence occurrence percentage between Part 1 and Part 2 of non-prior knowledge condition (n = 20, α = 0.05), t(18) = 0.93, p = 0.18; not significant.

To compare the difference between Part 1 and Part 2 of each condition, we examine the difference in mutual gaze convergence between Part 1 and Part 2 of each condition using paired T-test analysis (α = 0.05). The paired T-test result shows that the occurrence percentage of mutual gaze convergence in Part 1 of prior knowledge condition is (highly) significantly higher than the occurrence percentage of mutual gaze convergence in Part 2, t(18) = 4.48, p < 0.0001, as shown in Fig. 5(a). For non-prior knowledge condition, the T-test result demonstrates that there is no significant difference in mutual gaze convergence occurrence percentage between Part 1 and Part 2, t(18) = 0.93, p = 0.18, as shown in Fig. 5(b).

### Head nodding synchrony result

Head nodding synchrony is observed using accelerometers that attached on the lecturer’s and student’s forehead. We identified head nodding synchrony by calculating time lag of head motion between the lecturer and the student from their accelerometers data, using Spearman’s Rank Correlation. The data of 29 participants is analyzed due to data collection error in 1 of non-prior knowledge condition pair (10 pairs in prior knowledge condition and 9 pairs in non-prior knowledge condition, n = 19).

#### Total head nodding synchrony (Total)

We compared total head nodding synchrony occurrence percentage during the interaction as a whole between prior knowledge condition and non-prior knowledge condition using T-test analysis (α = 0.05), similar to mutual gaze convergence analysis. The result shows statistical significance in the occurrence percentage of total head nodding synchrony between prior knowledge condition and non-prior knowledge condition as illustrated in Fig. 3(b), t(16.67) = 1.83, p = 0.04. The result indicates that the interaction in prior knowledge condition establishes significantly higher head nodding synchrony than the interaction in non-prior knowledge condition. The average percentages of total head nodding synchrony of prior knowledge condition and non-prior knowledge condition are 4.59 and 3.20, respectively.

#### Head nodding synchrony in Part 1 (Part 1)

**Figure 6:**
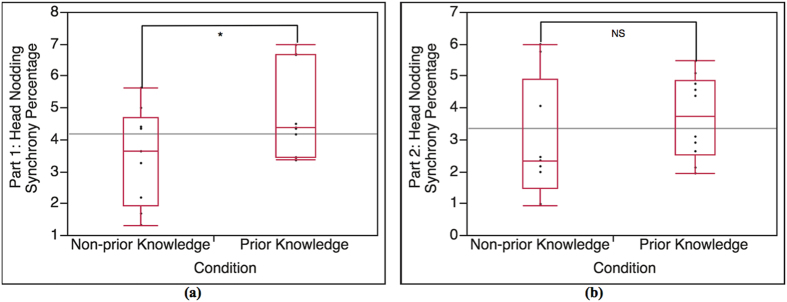
Analysis result of head nodding synchrony for Part 1 and Part 2. **(a)** T-test result between Part 1 of prior knowledge condition and Part 1 of non-prior knowledge condition (n = 19, α = 0.05), t(16.57) = 1.92, p = 0.03; significant. **(b)** T-test result between Part 2 of prior knowledge condition and Part 2 of non-prior knowledge condition (n = 20, α = 0.05), t(13.98) = 0.97, p = 0.17; not significant.

The result data of each pair is divided into 2 parts according to the lecture content. We conducted cross-condition T-test analysis on the result of Part 1 (α = 0.05). The cross-condition T-test result of head nodding synchrony occurrence percentage in Part 1 is shown in Fig. 6(a). The result indicates that head nodding synchrony occurrence percentage in Part 1 of prior knowledge condition is significantly higher than Part 1 of non-prior knowledge condition, t(16.57) = 1.92, p = 0.03. The average percentages in Part 1 of prior knowledge condition and non-prior knowledge condition are 4.80 and 3.50, respectively.

#### Head nodding synchrony in Part 2 (Part 2)

In case of Part 2 (baseline), we also expected no statistical significance between the 2 conditions. T-test result shows no statistical significance in Part 2 between prior knowledge condition and non-prior knowledge as expected, t(13.98) = 0.97, p = 0.17 (α = 0.05), as shown in Fig. 6(b). The average percentages in Part 2 of prior knowledge condition and non-prior knowledge condition are 3.7 and 2.97, respectively.

#### Head nodding synchrony between Part 1 and Part 2 of each condition (Part 1-Part 2)

**Figure 7:**
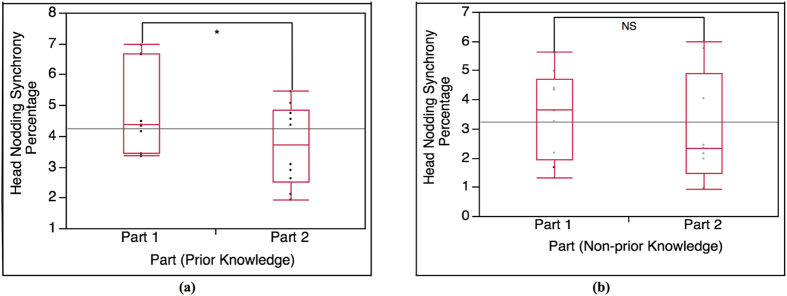
Analysis result of head nodding synchrony occurrence percentage between Part 1 and Part 2 of each condition. **(a)** Paired T-test result of head nodding synchrony occurrence percentage between Part 1 and Part 2 of prior knowledge condition (n = 19, α = 0.05), t(18) = 1.8, p = 0.04; significant. **(b)** Paired T-test result of head nodding synchrony occurrence percentage between Part 1 and Part 2 of non-prior knowledge condition (n = 19, α = 0.05), t(16) = 0.66, p = 0.26; not significant.

To compare the difference between Part 1 and Part 2 of each condition, we examine the difference of head nodding synchrony between Part 1 and Part 2 of each condition using paired T-test analysis (α = 0.05). The paired T-test result shows that the occurrence percentage of head nodding synchrony in Part 1 of prior knowledge condition is significantly higher than the occurrence percentage of head nodding synchrony in Part 2, t(18) = 1.8, p = 0.04, as shown in Fig. 7(a). In case of non-prior knowledge condition, the T-test result shows that there is no significant difference in head nodding synchrony occurrence percentage between Part 1 and Part 2, t(16) = 0.66, p = 0.26, as shown in Fig. 7(b).

### Ratio of Change

In addition, we calculated the decrease ratio of mutual gaze convergence and head nodding synchrony occurrence percentage of Part 1 to Part 2 in both prior knowledge and non-prior knowledge condition.

#### Ratio of change in mutual gaze convergence (Ratio-mutual gaze convergence)

**Figure 8:**
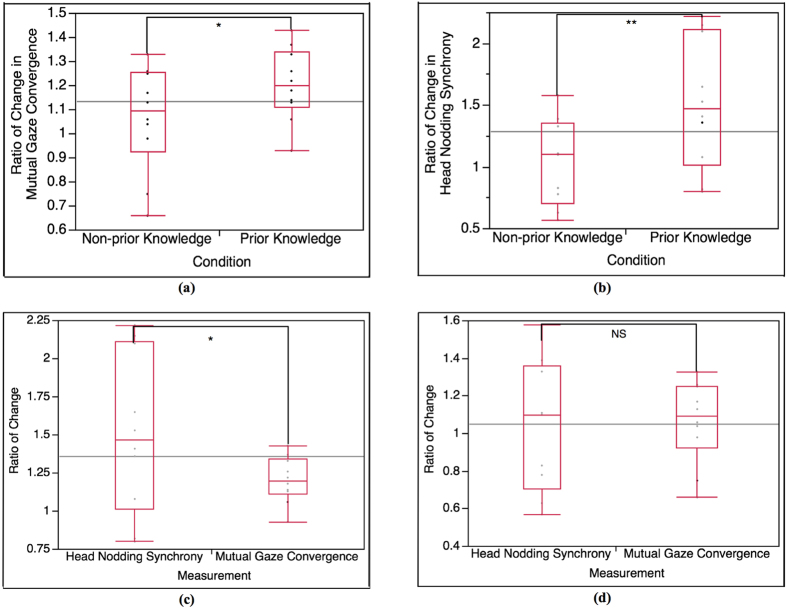
Analysis result of change ratio of Part 1 to Part 2. **(a)** T-test result of change ratio in mutual gaze convergence between prior knowledge condition and non-prior knowledge condition (Ratio-mutual gaze convergence), t(15.98) = 1.69, p = 0.05 (n = 20, α = 0.05); marginal significant. **(b)** T-test result of change ratio in head nodding synchrony between prior knowledge condition and non-prior knowledge condition (Ratio-head nodding synchrony), t(15.86) = 2.34, p = 0.01 (n = 19, α = 0.05); highly significant. **(c)** T-test result of change ratio between mutual gaze convergence and head nodding synchrony in prior knowledge condition (Ratio-between measurement), t(10.47) = 1.78, p = 0.05 (n = 20, α = 0.05); marginal significant. **(d)** T-test result of change ratio between mutual gaze convergence and head nodding synchrony in non-prior knowledge condition (Ratio-between measurement), t(13.03) = 0.2, p = 0.57 (n = 19, α = 0.05); not significant.

The average change ratio of mutual gaze convergence occurrence percentage of Part 1 to Part 2 in prior knowledge condition is 1.21 while the average change ratio of mutual gaze convergence occurrence percentage in non-prior knowledge condition is 1.06. We further conducted T-test analysis to see how significant of the change ratio between the 2 conditions (α = 0.05). The t-test result shows that the ratio of change of Part 1 to Part 2 in prior knowledge condition is (marginal) significantly higher than in non-prior knowledge condition, t(15.98) = 1.69, p = 0.05 (Fig. 8(a)).

#### Ratio of change in head nodding synchrony (Ratio-head nodding synchrony)

To perceive the change ratio of Part 1 to Part 2 of head nodding synchrony occurrence percentage in both prior knowledge and non-prior knowledge condition, we also calculated the change ratio from both conditions as well. The average change ratio of head nodding synchrony occurrence percentage in prior knowledge condition is 1.51, and 1.05 for non-prior knowledge condition. The T-test analysis indicates that the ratio of change in prior knowledge condition is (highly) significantly higher than in non-prior knowledge condition, t(15.86) = 2.34, p = 0.01 (α = 0.05), as shown in Fig. 8(b).

#### Ratio of change between 2 behavioral measurements (Ratio-between measurements)

To examine the degree of change between the two behavioral measurements in both conditions, we compared the change ratio of Part 1 to Part 2 between mutual gaze convergence and head nodding synchrony occurrence percentage using T-test analysis (α = 0.05). In prior knowledge condition, the T-test result shows that the change ratio of Part 1 to Part 2 of head nodding synchrony is (marginal) significantly higher than the change ratio of mutual gaze convergence, t(10.47) = 1.78, p = 0.05, as shown in Fig. 8(c). In non-prior knowledge condition, the T-test result indicates that the change ratio of Part 1 to Part 2 between the 2 behavioral measurements shows no significant difference, t(13.03) = 0.2, p = 0.57, as shown in Fig. 8(d).

## Discussion

According to the result of the interaction between the lecturers and the students in prior knowledge condition and non-prior knowledge condition (Total), it shows that the difference in occurrence percentage of total mutual gaze convergence and head nodding synchrony between the 2 conditions is statistically significant (Fig. 3). The lecturers and the students with prior knowledge (prior knowledge condition) establishes higher mutual gaze convergence and head nodding synchrony than the lecturers and the students with no prior knowledge (non-prior knowledge condition). The result of Total is also supported by the result of Part 1 (highly related to prior knowledge). The Part 1 result shows the same tendency as the Total result (Figs 4(a) and 6(a)). Since Part 1 of the lecture content is highly related to the prior knowledge, the result of Part 1 reveals that the interaction in prior knowledge condition also establishes significantly higher occurrence percentage of mutual gaze convergence and head nodding synchrony than the interaction in non-prior knowledge condition. In term of the students’ nonverbal behaviors (straight gaze toward the lecturer and head nodding), the results of Total and Part 1 shows that student’s prior knowledge can influence their own nonverbal behaviors. According to prior knowledge and Ready State definition, the students who are at Ready State (prior knowledge condition) can proceed their learning and understanding throughout the lecture by creating knowledge linkage between their prior knowledge and the new knowledge easier than those students who are not at Ready state (non-prior knowledge condition), which helps enhancing the students’ understanding and attentiveness during class. To measure such internal state, many researchers asserted that nonverbal indicators that represent individual’s feedback of attentiveness, understanding or acceptance are gaze and head nodding^30^,^31^,^32^. With the definition of Ready State together with the supported nonverbal indicators, it can be implied that the students in prior knowledge condition have higher possibility of establishing more eye contact and head nodding feedback to the lecturer during the interaction. In the case of nonverbal-interactional behaviors (mutual gaze convergence and head nodding synchrony) between the lecturer and the student, this phenomenon can be explained by interactional expectation^33^ and conversational grounding theory^34^. The interactional expectation theory indicates that one’s action can affect another’s and that individual’s expectations of others’ behaviors can cause behavioral changes during the interaction. Conversational grounding theory also supports that speakers expect frequent and incremental feedback from listeners, which can be in form of making and breaking gaze or nodding^35^. With grounding feedback provided by the listeners, the speakers can proceed the conversation with greater sense of rapport and confidence. During the interaction, the speakers expect the listeners’ gaze direction to be at them, and head nodding as the feedback response of understanding and that the listeners are with the speakers. Such high nonverbal feedback behaviors from the students in prior knowledge condition can influence the lecturers’ behaviors and encourage the lectures to have more eye contact and gesture movement in order to be in sync with each other^29^. More feedback from the students in prior knowledge condition might also meet the lecturers’ expectation. As the result, it might invoke more rapport from the lecturers. Furthermore, more rapport from the lecturers can help maintaining and enhancing the student’s attention and interest^19^,^36^,^37^ that can create the loop of conversational grounding between the lecturers and the students. This leads to higher chance of forming mutual gaze convergence and head nodding synchrony in prior knowledge condition.

For Part 2, the comparison results between Part 1 and Part 2 of each condition (Part1-Part 2) for both mutual gaze convergence (Fig. 5(a,b)) and head nodding synchrony (Fig. 7(a,b)) affirm that Part 2 is a baseline for both conditions since the results in of Part 2 are consistent in both conditions. We then expected no statistical significance on the mutual gaze convergence and head nodding synchrony occurrence percentage in Part 2 between prior knowledge condition and non-prior knowledge condition as well. However, the result of mutual gaze convergence occurrence percentage also shows statistical significant between the 2 conditions while the result of head nodding synchrony occurrence percentage shows no significant difference as expected (Figs 4(b) and 6(b)). The interaction in prior knowledge condition still maintains significantly higher mutual gaze convergence during Part 2. Though the result of mutual gaze convergence and head nodding synchrony in Part 2 indicate divergence in statistical significance, it does shows the same tendency that both mutual gaze convergence and head nodding synchrony occurrence percentage in prior knowledge condition decrease. This finding can be supported by the results of change ratio of Part 1 to Part 2 of mutual gaze convergence (Ratio-mutual gaze convergence) and head nodding synchrony (Ratio-head nodding synchrony). The results of change ratio indicate that even though the change ratio of Part 1 to Part 2 of both mutual gaze convergence and head nodding synchrony between prior knowledge condition and non-prior knowledge condition demonstrates significant difference, the results reveal that the ratio of change in mutual gaze convergence is smaller than the ratio of change in head nodding synchrony (Fig. 8(a,b)). We, therefore, confirmed the statistical significance by examining the ratio of change between 2 behavioral measurements (Ratio-between measurements), which demonstrate the comparison between the ratio of change from Part 1 to Part 2 between mutual gaze convergence and head nodding synchrony (Fig. 8(c,d)). The results indicate significant difference in prior knowledge condition while showing no significant difference in non-prior knowledge condition. From these supporting results, we additionally discovered and affirmed that mutual gaze convergence and head nodding synchrony have different temporal range regarding to the influence of prior knowledge. In case of gaze, as previous studies asserted that gaze is the most significant, reliable, and observable indicator of interest^38^ and attentiveness^30^,^31^, many researchers also provide studies on various influence factors that can affect individual’s interest and attentiveness, for instance, degree of understanding and interactants’ characteristics. The study of Abrantes revealed that the higher degree of understanding or learning performance leads to a higher level of student’s interest^39^, which is supported by Tobias’s^6^ and Van’s^7^ studies. Furthermore, the study of Broz also supported that the characteristics of both does is not only influence the attentiveness of the interactants but it also directly affects on the mutual gaze convergence between the interactants^40^. These influence factors can cause prolongation of students’ interest and attention from Part 1 to Part 2, which help decelerating the declination of mutual gaze convergence, especially for the students with prior knowledge (prior knowledge condition) since the students in prior knowledge condition are all at Ready State, where they can proceed their learning and understanding throughout the lecture by creating knowledge linkage easier. Therefore, the level of understanding of the lecture content in the students with prior knowledge seems to be higher than the students with no prior knowledge, which might increase their interest in the lecture content from Part 1 to Part 2 and lead to the prolongation of straight gaze toward the lecturer. This can create a higher chance of forming mutual gaze convergence between the lecturers and the students. In case of head nodding, head nodding is considered as an indicator of coordination between the interactants^29^ and as a body language of understanding and acceptance^32^. The researchers revealed that head nodding will be expressed when coordination between the interactants is occurred and when the individual shows understanding or agreement. According to the results of head nodding synchrony, the influence factors that can affect individual’s gaze behaviors such as interest and attentiveness have no effect to head nodding behaviors. Low head nodding during the interaction in Part 2, therefore, indicates low coordination between the lecturers and the students and low understanding in the lecture content for the students, which leads to lower chance of forming head nodding synchrony.

Since Charles Darwin stated in his study that nonverbal behavior reveals emotion^32^, many researchers started to investigate and pay more attention on the relationship between various internal states or emotions and nonverbal behaviors. According to the previous studies, they discovered that individual’s nonverbal behaviors can convey and indicate individual’s emotions or internal states, for instance, anger^41^, love^42^, interest and attention^38^, attitudes and interpersonal styles^43^, trust^44^, and coordination between interactants^29^. By applying the concept of emotion-nonverbal behavior relationship from the previous studies, we can infer prior knowledge as internal state and since we interest in human-human interaction instead of individual’s nonverbal behavior, we infer the nonverbal-interactional behaviors, namely mutual gaze convergence and head nodding synchrony, as nonverbal behaviors between 2 interactants.

The findings in this study support our hypothesis and indicate that the interaction with prior knowledge will establish more mutual gaze convergence and head nodding synchrony, especially during Part 1 of prior knowledge condition. For future work, further investigation on potential order effect between Part 1 and Part 2 should be more concentrated since we cannot alter the order between Part 1 and Part 2 in this study due to the continuity of the content. Cultural and gender difference are also necessary to attain insight understanding on diversity in human behaviors and their possible influences.

In conclusion, this study evinces that more mutual gaze convergence and head nodding synchrony are significantly formed and observed in the interaction with prior knowledge. We further discovered that head nodding synchrony can clearly indicate the difference in prior knowledge. The occurrence percentage of head nodding synchrony is established differently in the interaction with or without prior knowledge. On the other hand, mutual gaze convergence is significantly affected by prior knowledge. The students’ understanding level can enhance and prolong the students’ interest and attentiveness from Part 1 to Part 2, which decelerates the declination of mutual gaze convergence occurrence percentage. Since mutual gaze convergence is significantly affected by prior knowledge, it has longer temporal range, compared to head nodding synchrony. Our study indicates that prior knowledge facilitates the occurrence of mutual gaze convergence and head nodding synchrony with different temporal range. Here, we can also infer that mutual gaze convergence and head nodding synchrony can be served as the indicators of the difference in prior knowledge. The interaction with/without prior knowledge can be evaluated by measuring mutual gaze convergence and head nodding synchrony.

## Methods

### Experiment

#### Task

The experiment task is face-to-face interaction between 2 individuals in short lecture task (approx. 5 minutes). Participants take the role of lecturer and student in each trial. The lecturer participant teaches the prepared article to the student participant. The protocols and procedures used in this experiment were approved by the Tokyo Institute of Technology’s Ethical Review Board for Epidemiological Studies. The methods were carried out in accordance with the approved guidelines.

#### Content

The article “Naps Clear Brain’s Inbox, Improve Learning” in Japanese version from National Geographic’s site is chosen as the lecture content in this experiment. The article describes the advantages of sleeping and napping on improving human’s learnability, especially in memorization and information recall.

#### Condition

This experiment consists of 2 conditions: prior knowledge and non-prior knowledge. In prior knowledge condition, the experimenter conducted the prior knowledge activating session by providing relevant information about the lecture content and assessed if the students are at Ready State before starting the lecture session while in non-prior knowledge condition, we conducted the lecture session regardless of the student’s prior knowledge.

#### Control Parameter

Prior knowledge is the control parameter in this study. We further divided the lecture content into 2 parts. The content in Part 1 contains the information about sleeping state that helps on memorization and how sleeping or napping enhance the brain activities on transferring short-term memory to long-term memory, which is correlated to the content in prior knowledge activating session. In prior knowledge activating session, the content describes how brain stores short-term memory and long-term memory, and the difference between short-term memory and long-term memory. In other words, the content in the Part 1 is organized to contain the information that is highly related to the information provided to the student in prior knowledge activating session. For Part 2, the content contains the information about the experiment on the relationship between sleeping or napping and memory, including the results of the experiment. Additionally, the content in Part 2 introduces new and different technical terms, for instance, “cache memory” instead of “short-term memory”. In other words, Part 2 is organized to contain the information that is rarely related to the information provided in prior knowledge activating session with different technical terms. Part 2 is also considered as the baseline content because the content in Part 2 contains the information that is unfamiliar to the participants in both conditions. With these 2 divided parts, we can observe the changes in mutual gaze convergence and head nodding synchrony across the part that is highly related and rarely related to the information in prior knowledge activating session and affirm the influence of prior knowledge toward mutual gaze convergence and head nodding synchrony.

#### Participants

All 30 participants are native Japanese student, age ranging from 21 to 47 years old. The participants were randomly grouped with the same gender into 10 triplets, 8 male triplets and 2 female triplets. The roles of each participant were also randomly assigned by the experimenter. The participant roles of each triplet are 1 lecturer and 2 students, 1 student in prior knowledge condition and another student in non-prior knowledge condition. The same lecturer conveyed the same lecture content to the student of both conditions as we can affirm that the teaching style of the lecturer is the same for each triplet. It is, therefore, 2 pairs in each triplet. In order to exclude consideration of familiarity effect between the participants, the interaction between participants before the experiment were not allowed. Informed consent was obtained from all participants before participating the experiment.

#### Equipment

2 web cameras, 2 glasses cameras, 1 notebook computer and 3 accelerometers were used in this experiment. The cameras and accelerometers specification is shown in Table 1.

#### Environmental setup

The environmental setup for each trail in this experiment is illustrated in Fig. 1. The lecturer and student were seated on the opposite side of each other with a table between them, 1.5 meters apart. Each web camera was set 50 cm apart from each participant, facing directly to the participant’s face. Each participant also had to wear a glasses camera to capture their partner’s face on the opposite side and attached the accelerometer on their forehead to observe their head motion during the interaction. In order to minimize the external factors that might affect the participants’ attention, we asked the participants to pay attention to each other during the interaction. For minimal saliency effects, there were neither experimenters nor movable objects presented in the experiment room during the interaction.

#### Procedure

The experiment consists of 2 main sessions for both conditions, lecturer training and lecturing session. Prior knowledge activating session is an additional session for prior knowledge condition.

The first session is lecturer training. All lecturer participants were asked to prepare and practice their teaching on the provided article before participating the experiment. In this session, the lecturers were trained and practiced with the experimenter the day prior to the experiment day and again before the experiment in order to ensure that they could give a smooth lecture. After training, we evaluated the lecturers’ lecture content delivery by using 10-question test regarding the main points of the lecture content to affirm that they could correctly convey through the lecture content sequence.

The second session is prior knowledge activating. This session is an additional session for prior knowledge condition only. In this session, the students in prior knowledge condition were trained by the experimenter. The experimenter provided the basic information about brain functions and memory. The content describes how brain stores short-term and long-term memory, and the difference between short-term memory and long-term memory, which is related to the lecture content in Part 1, in order to activate the prior knowledge of the students and to prepare the students to be at Ready State before continuing to lecture session. The experimenter also evaluated the students’ Ready State condition by assessing their understanding in the provided information via 10-question test.

The last session is lecturing. In this session, the lecturer conveyed the lecture content to the student.

### Mutual gaze convergence detection

As we only focus on gaze direction instead of precise gaze position, the detection methods and tools are simple. We use web cameras and glasses cameras as tools to capture interactants’ gaze behaviors. Mutual gaze convergence detection method consists of 5 main processes: eye detection, looking straight identification, partner face detection, gaze direction detection, and mutual gaze convergence inference, executing in sequence^45^.

#### Eye detection

**Figure 9:**
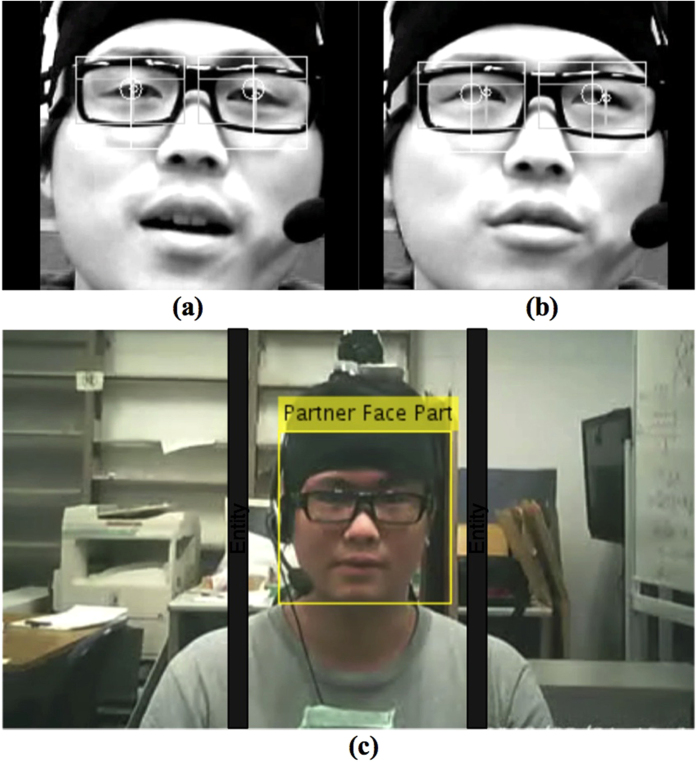
Process of mutual gaze convergence detection method screenshots. **(a)** Eye center localization (looking straight). **(b)** Eye center localization (looking elsewhere). **(c)** Partner face detection.

Web camera video files were manually divided into 2 parts according to the lecture content division and were analyzed one by one using eyelike project (OpenCV C ++) as a framework to perform eye detection. Starting with face detection, facial features are identified using *face_cascade* function to create face bounding box. Left and right eye regions are extracted from the detected face bounding box. Image gradients technique is applied to perform eye center localization in each eye region by locating the most gradient vectors intersection^46^. In addition, this process also performed straight gaze detection for each eye center using adaptive threshold in order to marginalize the effects of size difference and head movement of individuals. If the detected eye region size is less than 80 pixels, the threshold of looking straight is set to be ±8 pixels from the middle point of the detected eye region. For the >80 and ≤90 pixels, and the >90 pixels detected eye region size, the threshold is set to be ±12, and ±15 pixels, respectively. The adaptive threshold used in this process is referred from 8 training data set with at least 70% accuracy for all data set. If the detected eye center is within the threshold, we assume that the individual is looking straight in that particular frame (Fig. 9(a)). Otherwise, looking elsewhere (Fig. 9(b)). The final products of this process are: looking straight or looking elsewhere of left and right eye for each frame.

#### Looking straight identification

Though ordinary human eyes have symmetry in line of sight, 2 separated eyes comparison is required to compensate the accuracy of the eye detection result. This process compares the left and right eye detection results. If at least one side of the eyes is detected as looking straight, we considered that the individual is looking straight in that particular frame. For easier further comparison, we converted looking straight identification result from 30 frames to 1 second unit, as the cameras’ frame rate is 30 fps, by assigning the most occurrence from each 30 frames. The final products of this process are: looking straight or looking elsewhere for each second.

#### Partner face detection

Glasses camera video files of each individual is executed in this process. We executed the partner face detection using MATLAB’s *vision.cascadeObjectDetector* function to detect if the partner face is located within the middle of the video frame threshold or not. The seat position and video resolution size were fixed. We, thus, can infer to the middle of the frame easily. The threshold used for this process is referred from the 8 training data set with at least 70% accuracy for all data set. According to the training data sets, we observed that, in average, when the participant is looking straight to the partner, the partner face is detected not exceeding ±100 pixels from the middle of the frame. The ±100 pixels threshold from the middle of the frame is set (Fig. 9(c)). If the detected partner face is located with in the threshold of that particular frame, we assume that the individual is facing their head directly toward their partner direction. For easier further comparison, 30-frame-to-1-second conversion is performed similarly to the looking straight identification process. The final products of this process are: partner face detected or not detected for each second.

#### Gaze Direction Detection

Gaze direction detection performs the comparison between looking straight identification result and partner face detection result of each individual. If both results or looking straight identification result are positive, we consider that the individual is having straight gaze direction toward their partner in that particular second. The final products of this process are: straight gaze direction or non-straight gaze direction for each second.

#### Mutual Gaze Convergence Inference

The last process compares gaze direction detection results of the two individuals, the lecturer and the student participant, second by second. If both gaze direction detection results reveal that they are both having straight gaze direction at each other, we assume this scenario as mutual gaze convergence, and non-mutual gaze convergence, otherwise. The final products of this process are: mutual gaze convergence or non-mutual gaze convergence for each second.

### Head nodding synchrony detection

The accelerometers were connected to the notebook computer via Bluetooth and were operated and recorded by SyncRecord Software (ATR Promotions Co., Ltd.). Head nodding synchrony detection method consists of 5 main processes: head motion acceleration norm calculation, time-frequency of acceleration norm analysis, amplitude spectrum of head motion extraction, head motion synchrony identification, and head nodding synchrony identification^47^.

#### Head motion acceleration norm calculation

In this process, we calculated the norm |a(t)| from axial directions (x, y, z) as follow:


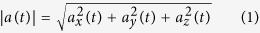


**Figure 10:**
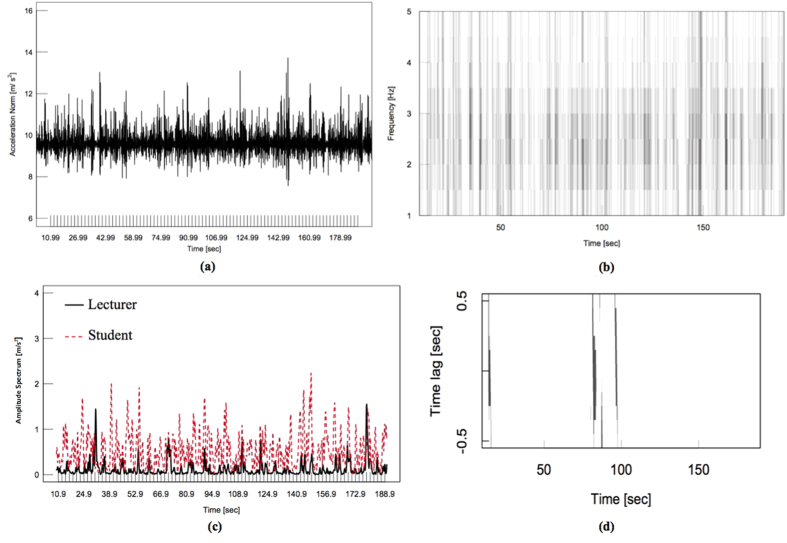
Process of head nodding synchrony detection method. **(a)** Head motion acceleration norm calculation result. **(b)** Time-frequency of acceleration norm analysis result. **(c)** Amplitude spectrum of 3.0 Hz. from head motion result from of the lecturer and the student. **(d)** Head motion synchrony identification result of lecturer-student comparison.

Time resolution was set to 0.01 seconds. The example of this process result is illustrated in Fig. 10(a). According to the figure, Head motion behaviors are represented as high peaks.

#### Time-frequency of acceleration norm analysis

Once the acceleration norm is calculated, Short-time Fourier Transform (STFT) is applied using a Humming window ω(t) as a window function. The window width is set to 1.28 seconds. STFT of head motion acceleration norm was calculated using the following equation.


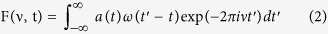


Where *v* = frequency in Hz, *ω*(*t*) = Humming window function, t = central time of window function. The window moving time is set to be 0.1 seconds for calculating the Fourier Transform. By applying Linear Interpolation with respect to frequency direction *v*, the data of frequency direction is calculated, which shown in Fig. 10(b). The frequency band is ranging from 1.0 to 5.0 Hz. The darker shade represents the higher amplitude spectrum, which can be inferred to higher intensity of head motion.

#### Amplitude spectrum of head motion extraction

Once the result of STFT is obtained, this process performs extraction of every 0.5 Hz. amplitude spectrum [1.0, 1.5, 2.0, 2.5, 3.0, 3.5, 4.0, 4.5, 5.0 Hz.]. With this process, we obtained the interactants’ head motion amplitude spectrum. The amplitude spectrum will be further compared with their partner’s head motion amplitude spectrum in order to indicate the head motion synchrony in the next process. Figure 10(c) shows and the example of amplitude spectrum extraction of 3.0 Hz.

#### Head motion synchrony identification

This process calculates time lag of head motion between lecturer and student using Spearman’s Rank Correlation to detect the head nodding synchrony. It had been reported that synchronization between 2 participants in tapping task, is only possible for the temporal interval of tapping sounds within a range of 200 ms to 1800 ms^48^. Therefore, in this study, the window width is set to 1.8 seconds. The frame shift of the window is set to 0.1 seconds. The time lag of ±0.5 seconds is used with a temporal interval of 0.1 seconds since it has been reported that the therapist’s body movements, in positive psychotherapeutic session between a client and therapist, occur with a 0.5 seconds delay compared with the clients^49^. Furthermore, it was also reported that the synchrony between an infant’s movement and adult speech occurred at a time lag of 0.05 ± 0.2 seconds^50^. The criteria of head motion synchrony identification are, firstly, the rank correlation of Spearman is positively significant. Secondly, the average of amplitude spectrum of individual’s head motion is at least 90% of the total amplitude spectrum. Figure 10(d) demonstrates the example result of time lag calculation of head motion synchrony. The head motion synchrony is represented with the black vertical lines.

#### Head nodding synchrony identification

Since head nodding is a series of consecutive up-down head motion, this process extracts only head nodding synchrony from head motion synchrony result by extracting the consecutive head motion synchrony that last longer than 0.5 seconds from every frequency band in order to distinguish between head nodding synchrony and head motion synchrony.

## Additional Information

**How to cite this article**: Thepsoonthorn, C. *et al*. Prior Knowledge Facilitates Mutual Gaze Convergence and Head Nodding Synchrony in Face-to-face Communication. *Sci. Rep.* **6**, 38261; doi: 10.1038/srep38261 (2016).

**Publisher's note:** Springer Nature remains neutral with regard to jurisdictional claims in published maps and institutional affiliations.

## Figures and Tables

**Table 1:** Experimental equipment specifications.

Specification	Web Camera	Glasses Camera	Accelerometer
**Model**	SANWA Supply CMS-V35BK, Japan	SPYDER E231, Japan	TSND 121, ATR Promotion, Japan
**Resolution**	2048 × 1536 pixels	1280 × 720 pixels	—
**Rate**	30 frames per second	30 frames per second	100 Hz
